# SARS-CoV-2 Transmission from Presymptomatic Meeting Attendee, Germany

**DOI:** 10.3201/eid2608.201235

**Published:** 2020-08

**Authors:** DirkJan Hijnen, Angelo Valerio Marzano, Kilian Eyerich, Corine GeurtsvanKessel, Ana Maria Giménez-Arnau, Pascal Joly, Christian Vestergaard, Michael Sticherling, Enno Schmidt

**Affiliations:** Erasmus MC University Medical Center Rotterdam, Rotterdam, the Netherlands (D. Hijnen, C. GeurtsvanKessel);; Fondazione IRCCS Ca' Granda Ospedale Maggiore Policlinico, Milan, Italy (A.V. Marzano);; Università degli Studi di Milano, Milan (A.V. Marzano);; Karolinska Institutet, Solna, Sweden (K. Eyerich);; Universitat Autònoma de Barcelona, Barcelona, Spain (A.M. Giménez-Arnau);; INSERM U—Rouen University Hospital, Rouen, France (P. Joly);; Aarhus University Hospital, Aarhus, Denmark (C. Vestergaard);; University Hospitals Erlangen, Erlangen, Germany (M. Sticherling);; Lübeck Institute of Experimental Dermatology, Lübeck, Germany (E. Schmidt);; University of Lübeck, Lübeck (E. Schmidt)

**Keywords:** COVID-19, SARS-CoV-2, severe acute respiratory syndrome coronavirus 2, viruses, respiratory infections, zoonoses, transmission, handshake, aerosolization, face-to-face contact, Germany, coronavirus disease

## Abstract

During a meeting in Munich, Germany, a presymptomatic attendee with severe acute respiratory syndrome coronavirus 2 infected at least 11 of 13 other participants. Although 5 participants had no or mild symptoms, 6 had typical coronavirus disease, without dyspnea. Our findings suggest hand shaking and face-to-face contact as possible modes of transmission.

We describe efficient spread of severe acute respiratory syndrome coronavirus 2 (SARS-CoV-2) resulting from contact with a presymptomatic infected person during a scientific advisory board meeting held February 20–21, 2020, in Munich, Germany; the country had <20 diagnosed coronavirus disease (COVID-19) cases at the time. Eight dermatologists from 7 countries and 6 scientists from the same company attended the meeting at a hotel in central Munich. The meeting was held in a room (≈70 m^2^) with conventional radiators; a U-shaped setup of tables were separated by a central aisle >1 m wide. During the meeting, refreshments were served buffet style in the same room 4 times. In addition to 9.5 hours of discussions, the participants had dinner on February 20 in a nearby restaurant. Additional direct contacts between participants were handshakes during welcome and farewell with few short hugs without kisses. None of the participants, including the index patient (participant [Pt] 1), showed any signs of infection (e.g., coughing, sneezing, respiratory symptoms, shivering, fever) before or during the meeting. No one wore a mask during the meeting. After the meeting, the index patient (Pt 1) shared a taxi with Pt 2, 4, and 9 for ≈45 min. 

After returning home the evening of February 21, Pt 1 sought care for fever. Reverse transcription PCR was performed on throat and nasal swab specimens, and SARS-CoV-2 RNA was detected by established methods (*1*). The patient was admitted to the hospital for supportive care, although he had only moderate symptoms (Table).

**Table:** Characteristics of all meeting participants, including face-to-face contact with the index patient, SARS-CoV-2 test results, symptoms and illness severity, isolation measures, and further contacts*

Pt no.	Country of origin	Contact with index patient†	PCR results	Other test results	Age, y/sex	Signs/symptoms	PGA (date)‡	COVID-19 severity§	Isolation measures	Family/other contacts
Pt 1	Italy	Index patient	Feb 22, positive; Mar 9, negative	NT	57/M	Feb 22–Mar 5, fever, coughing, sneezing, loss of smell and taste. Exanthema developed on day 7	5 (Feb 25)	Moderate	Feb 23–Mar 9, hospital isolation; Mar 9–23, home isolation	Family: 50/F tested negative; Feb 23–Mar 6, home isolation. Others: 4 hospital clinical staff members tested positive; 80 other contacts in 14-d home isolation, all tested negative
Pt 2	Spain	Dinner and taxi	Feb 27, positive; Mar 13, negative	NT	58/F	Feb 25–Mar 10, slight coughing, fever (for 1 d), GI symptoms, partial loss of smell	9.5	Mild	Feb 27–Mar 12, hospital isolation; Mar 13–27, home isolation	Family: Husband (59/M) tested positive Feb 28, asymptomatic; mother (86/F) tested positive Mar 4. Others: clinical staff member (F) tested positive, fever and headache; >10 others in home isolation, tested negative
Pt 3	Denmark	Neighbor during meeting	Feb 29, positive	NT	49/M	None	10	Asymptomatic	Feb 27–Mar 13, home isolation	Family: 45/F, 19/M, 15/F, all tested negative. Others: 12 contacts tested negative
Pt 4	France	Dinner	Feb 27, positive; Mar 8, negative	NT	60/M	Feb 24–26, headache, slight coughing	8.5 (Feb 25)	Mild	Feb 29–Mar 2, hospital isolation; Mar 2–10, home isolation	Family: 62/F tested negative. Others: 1 contact tested positive
Pt 5	Germany	Dinner	Feb 28, positive; Mar 6, negative; Mar 9, negative	ELISA (Euroimmun): Mar 10, positive for IgA and IgG	50/M	Feb 28–Mar 3, slight coughing, slight weakness; no fever	9.5 (Mar 2)	Mild	Feb 27–Mar 9, home isolation (with family)	Family: 46/F, 12/F, 11/F, 9/F, 9/M. 46/F tested negative Mar 6; ILI developed, tested positive Mar 13; severe headache, limb pain, high fever, and dyspnea thereafter for 5 d. Others: 1 contact tested negative, 1 contact tested positive
Pt 6	Sweden	Neighbor during meeting	NT	ELISA (Euroimmun): Mar 19, positive for IgA and IgG	40/M	None	10	Asymptomatic	Feb 29–Mar 7, hotel room isolation	Family: 41/F, 10/M, 9/F, all tested negative Mar 2. Others: no others tested
Pt 7	Germany	No	Feb 27, positive; Mar 16, negative; Mar 17, negative	NT	61/M	Feb 24–Mar 18, headache, slight coughing, weakness, loss of smell and taste	6 (Mar 28)	Moderate	Feb 27–Mar 12, hospital isolation; Mar 13–, home isolation	Family: 56/F, 24/F tested negative, home isolation 14 d. Others: 41 contacts in home isolation after contact (14 d); all tested negative
Pt 8	The Netherlands	No	Mar 1, 5, 9, positive; Mar 12, negative	ELISA (Euroimmun): Mar 7, 18, positive for IgA, neg for IgG	45/M	Feb 24 (only), ILI symptoms, fatigue. Feb 24–early April, loss of smell	6 (Feb 24)	Moderate	Feb 27–Mar 8, home isolation (with family)	Family: 43/F, 13/M and 14/M tested positive (home isolation Feb 29–Mar 8); 43/F ILI symptoms (Feb 27–29), 13/M asymptomatic, 14/M ILI symptoms Feb 25 only. 9/F tested negative (home isolation Feb 29–Mar 11); Others: no others tested or in isolation
Pt 9	Germany	Taxi	NT	NT	59/M	None	10	Asymptomatic or NA	Feb 27–Mar 9, home isolation	Family; no symptoms, NT. Others: no others tested or in isolation
Pt 10	Sweden	No	Feb 27, positive	NT	49/F	Feb 24–Mar 10, ILI symptoms, GI symptoms, nausea	5 (Feb 26)	Moderate	Feb 27–Mar 5, hospital isolation; Mar 5–18, home isolation	Family: 55/M, 19/F; no symptoms, NT. Others: unknown
Pt 11	Germany	Dinner	Feb 28, negative	NT	36/F	None	10	NA	Feb 26–Mar 8, home isolation (with family)	Family: 38/M tested negative. Others: unknown
Pt 12	Germany	No	Feb 27–Mar 8, positive 6 times; Mar 10–12, negative 2 times	NT	33/F	Feb 23–Mar 1, ILI symptoms, headache and limb pain, weakness, sore throat (mild), loss of appetite; loss of smell and taste for 7 more weeks	3 (Feb 24)	Moderate	Feb 26–Mar 10, hospital isolation; Mar 10–12, home isolation	Family: 33/M tested negative Feb 26/27, Mar 1; home isolation Feb 26–Mar 7. Others: 2 contacts in home isolation until Mar 7; both remained without symptoms, 1 tested negative
Pt 13	Germany	No	Feb 27, positive	NT	40/M	Feb 23–Mar 8, ILI symptoms (cough, limb pain; fever (39.5°C) days 1–4, followed by increased temperature days 5–9	5 (Feb 24)	Moderate	Feb 26–Mar 4, hospital isolation; Mar 4–9, home isolation (with family)	Family: 42/F, 11/F, both had increased temperature for 1 d (Mar 1); 11/F tested positive Mar 2), negative Mar 6/9; 42/F tested negative (Mar 2); isolation ongoing even after 2nd negative test. Others: unknown
Pt 14	Germany	No	Feb 27, positive; Mar 5, negative; Mar 6, negative	NT	45/F	Feb 23–Mar 6, headache and limb pain, fatigue; no fever	5 (Feb 27)	Moderate	Feb 27–Mar 7, hospital isolation	Family: 37/F, no symptoms, tested negative (Feb 27–Mar 7). Others: 15 contacts in home isolation; 14/15 no symptoms, 1 contact had symptoms develop and tested positive

*Last updated on April 20, 2020. All dates are for 2020. GI, gastrointestinal; ILI, influenza-like; NA, not applicable; NT, not tested; PGA, patient global assessment; Pt, participant. †Face-to-face contact with the index patient (Pt1) lasting >5 min. ‡Severity of COVID-19 symptoms quantified by the patients related to the time with the most severe symptoms from 1–10 (1, close to death; 10, full health). §Mild, PGA ≥8; moderate, PGA <8 but no hospitalization required.

National authorities contacted most meeting participants on February 26 (Pt 7, 11–13) and 27 (Pt 2–6, 8–12); Pt 14 was contacted by a coworker. Twelve participants, including Pt 1, were tested for SARS-CoV-2 by PCR; 2 were not, Pt 9 because he showed no signs of infection and Pt 6 because testing was not available at his location (New York, NY, USA) at the time. Pt 6 later underwent ELISA testing, which showed IgA and IgG against the recombinant S1 domain of structural protein of SARS-CoV-2 (Euroimmun, https://www.euroimmun.com) (*2*). Excluding the index patient, in 10/11 tested participants, SARS-CoV-2 RNA was detected. In 1 participant, Pt 11, the PCR result for SARS-CoV-2 RNA was negative (Table). Thus, the index patient infected >11 (85%) of the 13 other participants.

All participants were isolated either in a hospital or at home with or without their families, regardless of the outcome of the first PCR test. These measures resulted in the subsequent infection of 14 additional persons (Table). Of the 12 infected participants, 2 (17%) had no symptoms, 3 (25%) experienced mild influenza-like symptoms, and 7 (58%) experienced a considerable reduction of their health, without dyspnea, classified as moderate COVID-19 (Table). None of the participants had a relevant medical history.

The index patient (Pt 1) was most likely infected by an outpatient he had examined in Milan, Italy, on February 18. The index patient reported that he had experienced no symptoms when attending the meeting. Probable transmission of SARS-CoV-2 from presymptomatic persons has been reported (*3*,*4*), with viral load levels in the nose similar to those of symptomatic patients (*5*). In contrast to severe acute respiratory syndrome coronavirus and influenza virus, the infectiousness of SARS-CoV-2 peaks on or before symptom onset (*6*).

The exact mode of transmission during the meeting remains elusive. At least 4 routes have been suggested: droplets during face-to-face contacts, aerosolized droplets (<5 µm) via air flow, fomites, and hand shaking (*4*,*7*–*9*). We identified face-to-face contacts lasting >5 min with the index patient and the 11 infected participants during 2 lunches (30 min each), 2 coffee breaks (15 min each), and the social dinner (sitting close to Pt 2, 4, 5, and 11). We also tracked Pt 1 sitting next to Pt 3 and Pt 6 during the meeting, and a 45-min taxi ride after the meeting (with Pt 2, 4, and 9) (Table). The index patient sat ≈2.60 m away from the closest participant opposite to him and had an average talk time during the meeting. Virus aerosolization in the relatively small room that was heated by conventional radiators appears to be possible in light of the duration of the meeting. Transmission via fomites appears to be less likely because few objects (bottles, coffee pots, forks) were shared by all participants during the breaks. Telephone communication with hotel management on April 20 revealed that none of the involved hotel staff were tested for SARS-CoV-2 and no staff member reported symptoms consistent with COVID-19. 

Our findings indicate that hand shaking, aerosolization, and face-to-face contact may be relevant modes of transmission in this COVID-19 outbreak. Limitations include the lack of environmental samples and data about room ventilation and airflow patterns, as well as missing information about the infection status of Pt 9 and the inability to determine the actual impact of SARS-CoV-2 transmission from handshakes, droplets, and aerosolization.
